# Complete genome of *Arthrobacter alpinus* strain R3.8, bioremediation potential unraveled with genomic analysis

**DOI:** 10.1186/s40793-017-0264-0

**Published:** 2017-09-06

**Authors:** Wah-Seng See-Too, Robson Ee, Yan-Lue Lim, Peter Convey, David A. Pearce, Taznim Begam Mohd Mohidin, Wai-Fong Yin, Kok Gan Chan

**Affiliations:** 1 Division of Genetics and Molecular Biology, Institute of Biological Sciences, Faculty of Science, University of Malaya, Kuala Lumpur, Malaysia; 2 National Antarctic Research Centre (NARC), Institute of Postgraduate Studies, University of Malaya, 50603 Kuala Lumpur, Malaysia; 3 British Antarctic Survey, NERC, High Cross, Madingley Road, Cambridge, CB3 OET UK; 4 Faculty of Health and Life Sciences, University of Northumbria, Newcastle Upon Tyne, NE1 8ST UK; 5 Division of Microbiology, Institute of Biological Sciences, Faculty of Science, University of Malaya, Kuala Lumpur, Malaysia; 6 UM Omics Centre, University of Malaya, Kuala Lumpur, Malaysia; 7 Vice Chancellor Office, Jiangsu University, Zhenjiang, 212013 People’s Republic of China

**Keywords:** Cold active enzymes, Anti-freeze protein, Chitinase, Pyschrotolerant bacteria, Plant growth promoting bacteria

## Abstract

*Arthrobacter alpinus* R3.8 is a psychrotolerant bacterial strain isolated from a soil sample obtained at Rothera Point, Adelaide Island, close to the Antarctic Peninsula. Strain R3.8 was sequenced in order to help discover potential cold active enzymes with biotechnological applications. Genome analysis identified various cold adaptation genes including some coding for anti-freeze proteins and cold-shock proteins, genes involved in bioremediation of xenobiotic compounds including naphthalene, and genes with chitinolytic and *N*-acetylglucosamine utilization properties and also plant-growth-influencing properties. In this genome report, we present a complete genome sequence of *A. alpinus* strain R3.8 and its annotation data, which will facilitate exploitation of potential novel cold-active enzymes.

**Electronic supplementary material:**

The online version of this article (doi:10.1186/s40793-017-0264-0) contains supplementary material, which is available to authorized users.

## Introduction

The production of cold-adapted enzymes by psychrotolerant bacteria has important scientific and industrial interest due to their highly specific activity and catalytic efficiency at low and moderate temperatures [1]. The use of cold-adapted enzymes offers various advantages such as the reduction of undesirable chemical reactions that take place at high temperature, rapid enzymatic inactivation through thermal treatment, and reduction in energy demand required to fuel industrial processes at higher temperatures [2–4]. These beneficial traits are particularly useful in the development of sequential molecular biology processes, low temperature detergents, food and industrial bio-catalytic enzymes, and for bioremediation agents applicable during cold seasons and in cold regions. In this study, we perform complete genome sequencing on a psychrotolerant bacterium, 10.1601/nm.20084 strain R3.8 (=10.1601/strainfinder?urlappend=%3Fid%3DDSM+100969), originally isolated from soil collected from Rothera Point, Adelaide Island, maritime Antarctica. The optimum growth temperature range of this bacterium is 10–16 °C, which rendered it a promising source for discovery of novel cold-adapted enzymes. The complete genome sequence of 10.1601/nm.20084 strain R3.8 was generated using Single Molecule Real Time sequencing technology to provide a rapid and complete insight into its biotechnological potential. Here, we highlight various genome features that indicate the potential biotechnological value of 10.1601/nm.20084 strain R3.8 in the context of xenobiotic biodegradation and metabolism, chitin utilization, and as a potential component in bio-fertilizers.

## Organism information

### Classification and features

**Fig. 1:**
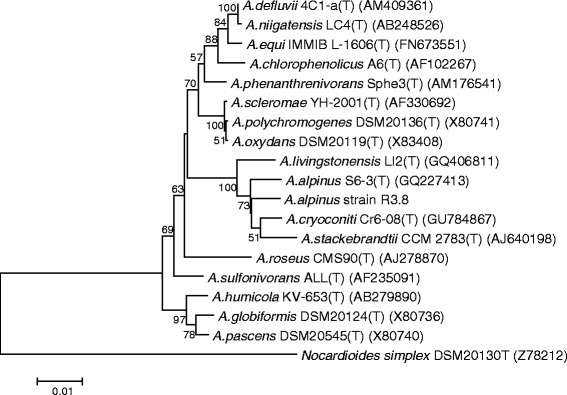
Neighbour-joining phylogenetic tree based on complete 16S rRNA gene sequences of *Arthrobacter alpinus* strain R3.8 and closely related species of the genus *Arthrobacter*. Bootstrap values (expressed as percentages of 1000 replications) are shown at the *branching points*. Bar, 1 nt substitutions per 100 nt

**Fig. 2:**
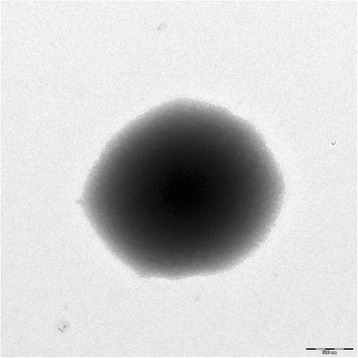
Transmission electron micrograph of *Arthrobacter alpinus* strain R3.8. The image was taken under a scanning transmission electron microscope (STEM, LIBRA 120; Carl Zeiss AG, Germany) at an operation voltage of 80 kV. The *scale bar* represents 500 nm

10.1601/nm.20084 strain R3.8, is a psychrotolerant soil bacterium originally isolated from a soil sample collected at Rothera Research Station, close to Antarctic Special Protected Area No.129 (68°07′S, 67°34′W). Strain R3.8 was isolated using basal medium supplied with C_6_-HSL as sole carbon source. An isolation temperature of 4 °C was used to select for psychrophilic or psychrotolerant bacteria maintained on Luria Bertani (LB) agar [5, 6]. The strain exhibited a 98.6% 16S rRNA nucleotide sequence similarity with 10.1601/nm.20084, the most phylogenetically closely related *Arthrobacter* species with standing in nomenclature (Fig. 1). The cells are Gram-positive, coccoid, and approximately 2.0 μM in width and 1.8 μM in length (Fig. 2). This pairwise 16S rRNA gene sequence similarity value suggested that strain R3.8 is 10.1601/nm.20084, following the species delineation threshold recommended by Stackebrandt and Ebers [7]. API test strips (API 20 E, API 20 E and API ZYM) incubated at 20 °C were used according to the manufacturer’s instructions to determine the physiological and biochemical characteristics as well as enzyme activities of strain R3.8. The results were compared with type strain of *A. alpinus* strain S6-3^T^. Strain R3.8 showed a closely similar biochemical profile with S6-3^T^ in all the API tests. Both strains did not produce catalase and cytochrome oxidase and were able to hydrolyze aesculin. Both strains were positive for activities of acidic phosphatase, esterase (C4), esterase lipase (C8), leucine arylamidase, α-glucosidase, ß-glucosidase, α-galactosidase, ß-galactosidase, ß-glucuronidase and α-mannosidase, and could utilizes D-glucose, lactose, L-arabinose, maltose, D-mannose, D-mannitol and *N*-acetylglucosamine as sole carbon source. Both strains were negative in indole production, H_2_S production and citrate utilization. Both were also negative for activities of arginine dihydrolase, lysine dihydrolase, ornithine dihydrolase, lipase (C14), *N*-acetyl-ß-glucosaminidase, trypsin, α-chymotrypsin, and α-fucosidase, and negative for the fermentation of glucose, mannitol, sucrose, inositol, sorbitol, rhamnose, melibiose, and amygdalin. However, strain R3.8 was not able to hydrolyze urea, unlike strain S6-3^T^, in both API 20 E and API 20 NE tests. In the API 20 NE test, strain R3.8 was positive for nitrate reduction, differing from strain S6-3^T^. In the API 20 E test, strain R3.8 was positive for fermentation of L-arabinose but strain S6-3^T^ was negative. In the API ZYM test, strain R3.8 did not produced alkaline phosphatase and naphthol-AS-BI- phosphohydrolase as produced by strain S6-3^T^.

Minimum Information about the Genome Sequence of 10.1601/nm.20084 strain R3.8 is summarized in Table 1.

**Table 1:** Classification and general features of *Arthrobacter alpinus* R3.8 according to the MIGS recommendations [34]

MIGS ID	Property	Term	Evidence code^a^
	Current classification	Domain Bacteria	TAS [35]
		Phylum *Actinobacteria*	TAS [36]
		Class *Actinobacteria*	TAS [37]
		Subclass *Actinobacteridae*	TAS [37, 38]
		Order *Actinomycetales*	TAS [37–40]
		Family *Micrococcaceae*	TAS [37–39, 41]
		Genus *Arthrobacter*	TAS [39, 42, 43]
		Species *Arthrobacter alpinus*	TAS [42]
		Strain R3.8	IDA
	Gram stain	Positive	TAS [42]
	Cell Shape	irregular rods, coccoid	IDA
	Motility	Non-motile	TAS [42]
	Sporulation	Non-sporulating	TAS [42]
	Temperature range	4–30 °C	IDA
	Optimum temperature	20–25 °C	IDA
	pH range; Optimum	6.0–9.0;7.0	IDA
	Carbon source	Acyl-homoserine lactone (AHLs),Yeast extract	IDA
MIGS-6	Habitat	soil	IDA
MIGS-6.3	Salinity	up to 10% NaCl (*w*/*v*)	IDA
MIGS-22	Oxygen requirement	Aerobic	TAS [42]
MIGS-15	Biotic relationship	Free living	NAS
MIGS-14	Pathogenicity	Non-pathogen	NAS
MIGS-4	Geographic location	Northern end of Rothera Point, adjacent to Antarctic Specially Protected Area 129, Antarctica	NAS
MIGS-5	Sample collection	December 2009	NAS
MIGS-4.1	Latitude	68°07′S	NAS
MIGS-4.2	Longitude	67°34′W	NAS
MIGS-4.4	Altitude	33 m	NAS

^a^Evidence codes – IDA: Inferred from Direct Assay; TAS: Traceable Author Statement (i.e., a direct report exists in the literature); NAS: Non-traceable Author Statement (i.e., not directly observed for the living, isolated sample, but based on a generally accepted property for the species, or anecdotal evidence). These evidence codes are from the Gene Ontology project

## Genome sequencing information

### Genome project history

The genome of 10.1601/nm.20084 strain R3.8 was sequenced to study its bioremediation properties, specifically focusing on naphthalene biodegradation. The assembled and annotated genome of 10.1601/nm.20084 strain R3.8 described in this paper has been deposited in GenBank (accession number of CP12677.1), the KEGG database (entry number of T04095) and the JGI portal with GOLD ID of Gp0124186 and IMG taxon ID of 2645727552. Sequencing, assembly and annotation of the complete genome were performed by the UM Omics Centre, University of Malaya, Malaysia. A summary of the project information is shown in Table 2.

**Table 2:** Project information

MIGS ID	Property	Term
MIGS 31	Finishing quality	Finished
MIGS-28	Libraries used	One library, PacBio 20-kb SMRTbell Library
MIGS 29	Sequencing platforms	PacBio RS
MIGS 31.2	Fold coverage	101.74X
MIGS 30	Assemblers	HGAP v. 2.2.0.p1 [40]
MIGS 32	Gene calling method	Prodigal 1.4, GeneMark, Glimmer v. 2.13
	Locus Tag	AOC05
	Genbank ID	CP012677.1
	GenBank Release Date	28-MARCH-2016
	GOLD ID	Gp0124186
	BioProject ID	PRNJA224116
MIGS 13	Source Material Identifier	DSM 100969
	Project relevance	Environmental, Biotechnological

### Growth conditions and genomic DNA preparation

10.1601/nm.20084 strain R3.8 was grown aerobically in 5.0 ml LB broth at 16 °C. A volume of 1.0 ml was then centrifuged at 2500 x g for 5 min at 4 °C and genomic DNA was extracted and purified using the MasterPure™ Gram positive DNA purification kit (Epicenter Technologies, USA) following the manufacturer’s instructions. The purity and quality of the genomic DNA obtained were assessed using a NanoDrop 2000 UV-Vis spectrophotometer (Thermo Scientific, USA) and quantified using Qubit 2.0 fluorometer (Life Technologies, MA, USA).

### Genome Sequencing and Assembly

The sheared genomic DNA of 10.1601/nm.20084 strain R3.8 was constructed into a 20 kb SMRTbell template library following the ‘Procedure and Checklist - 20 kb Template Preparation Using BluePippin^™^ Size-Selection System’ protocol [8, 9]. The purified and size-selected SMRTbell library was sequenced in five SMRT cells using P6C4 chemistry on a PacBio RS II sequencing system (Pacific Biosciences, USA). Sub-reads generated from the raw sequencing reads following adapter-removal were used as input data for de novo assembly using Hierarchical Genome Assembly Process version 2 [10]. The assembly of the 10.1601/nm.20084 strain R3.8 genome was based on 64,388 quality reads with a mean length of 7,335 bp resulting in a single circular chromosome consisting of 4,046,453 bp with 101.74-fold overall coverage.

### Genome Annotation

Gene prediction and annotation were performed using the Rapid Annotation Search Tool [11], Rapid Prokaryotic Genome Annotation [12] and NCBI Prokaryotic Genome Annotation Pipeline based on the best-placed reference protein set and GeneMarkS+. Additional gene identification was made using the KEGG database [13], Carbohydrate-Active Enzymes Database ([14], Pathosystem Resource Integration Center [15], and IMG ER [16].

### Genome Properties

**Fig. 3:**
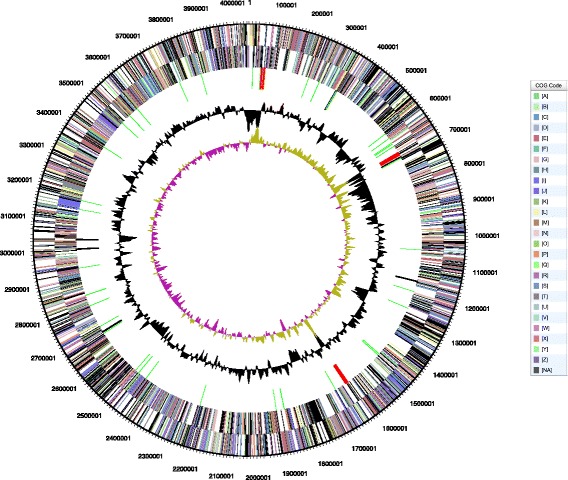
The genome graphical map of strain R3.8. From the outside to the center: genes on forward stand and genes on reverse strand (*color* by COG categories see legend), RNA genes (tRNAs *green*, rRNAs *red*, other RNAs *black*), GC content, GC skew

With 101.74 fold of coverage, the genome of 10.1601/nm.20084 strain R3.8 was assembled into a 4046,4553 bp circular chromosome with an average GC content of 62.2% (Table 3). No plasmid sequence was identified in this assembly (Table 1 and Fig. 3). A total of 3697 genes was predicted of which 3268 genes were identified as protein coding genes. A total of 69 RNA genes were also identified consisting of 18 rRNA (6 5S rRNA, 6 16S rRNA, and 6 23S rRNA) and 51 tRNA genes. 169 (5.17%) were designated as pseudo genes, 57 (1.74%) genes were frameshifted (Table 3). Furthermore, 61.54% of the predicted genes (3892) are represented by COG functional categories. Distribution of these genes and their percentage representation are listed in Table 4. The genome sequence is deposited in GenBank (accession number of CP12677.1), from which the genome sequence data can be accessed in the format of FASTA, annotated GenBank flat file, graphical and ASN.1 file.

**Table 3:** Genome statistics of *Arthrobacter alpinus* R3.8

Attribute	Value	% of Total
Genome size (bp)	4,046,453	100
DNA coding (bp)	3,578,529	88.44
DNA G + C (bp)	2,516,755	62.20
DNA scaffolds	1	100
Total genes	3892	100
Protein coding genes	3817	98.07
RNA genes	75	1.93
Pseudo gene	359	9.71
Genes in internal cluster	848	21.79
Genes with functional prediction	2870	73.74
Genes assigned to COGs	2395	61.54
Genes with Pfam domains	2987	76.75
Genes with signal peptides	101	2.60
Genes with transmembrane helices	942	24.20
CRISPR repeat	1

**Table 4:** Number of genes associated with general COG functional categories

Code	Value	% age	Description
J	180	6.69	Translation, ribosomal structure and biogenesis
A	1	0.04	RNA processing and modification
K	228	8.48	Transcription
L	112	4.17	Replication, recombination and repair
B	1	0.04	Chromatin structure and dynamics
D	25	0.93	Cell cycle control, Cell division, chromosome partitioning
V	53	1.97	Defense mechanisms
T	113	4.2	Signal transduction mechanisms
M	106	3.94	Cell wall/membrane biogenesis
N	28	1.04	Cell motility
Z	1	0.04	Cytoskeleton
W	3	0.11	Extracellular structures
U	25	0.93	Intracellular trafficking and secretion
O	104	3.87	Posttranslational modification, protein turnover, chaperones
C	183	6.81	Energy production and conversion
G	242	9	Carbohydrate transport and metabolism
E	281	10.45	Amino acid transport and metabolism
F	80	2.98	Nucleotide transport and metabolism
H	156	5.8	Coenzyme transport and metabolism
I	151	5.62	Lipid transport and metabolism
P	174	6.47	Inorganic ion transport and metabolism
Q	74	2.75	Secondary metabolites biosynthesis, transport and catabolism
R	228	8.48	General function prediction only
S	115	4.28	Function unknown
-	1497	38.46	Not in COGs

The total is based on the total number of protein coding genes in the genome

## Insights from the genome sequence

Functional annotation results of this genome are accessible from the complete genome directory of the KEGG ORGANISMS database with the organism prefix of aaq. Further, through the aaq hyperlink, cross-reference information is available in the form of protein, and small-molecules interaction network maps, BRITE biological systems hierarchical classifications, KEGG modules, and a whole genome map which can be visualized using genome map browser can be accessed through the subdirectory panel.

## Cold-adaptation genes

An antifreeze protein [AOC05_08780], a gene encoding a protein with ice-nucleation activity reported to be secreted by psychrotolerant bacterium into the surrounding medium at low temperatures to prevent the formation of ice crystals [17–19], was identified in the genome. Various temperature stress response genes were also identified. For example, the cold shock protein family that has been shown to allow bacterial response to rapid temperature shift, allowing bacteria cells to function to survive above their thermal optimum by serving as nucleic acid chaperones that may prevent the formation of secondary structures in mRNA at low temperature [20]. The NCBI locus tags for the cold shock proteins that were identified are AOC05_RS02130, AOC05_13125, and AOC05_RS01570.

## Biodegradation genes

Naphthalene is a group C (possible human carcinogen) benzenoid polycyclic aromatic hydrocarbon and is a pollutant widely encountered in nature [21, 22]. In 1990, naphthalene was recognized as one of the priority pollutants required to be controlled by the Environmental Protection Agency of the United States. In the genome of 10.1601/nm.20084 strain R3.8, various genes that are involved in naphthalene biodegradation were identified, including salicylate 1-monooxygenase [AOC05_08330], imidazole glycerol phosphate synthase cyclase [AOC05_05535] that is involved in 1- and 2-methylnaphthalene degradation, and other genes involved in 1,4-dichlorobenzene degradation, namely enoyl-CoA hydratase [AOC05_00980, AOC05_05365, AOC05_11215, AOC05_11270, AOC05_11290, AOC05_11310, AOC05_13700, AOC05_13710], alkaline phosphatase [AOC05_01425, AOC05_03310, AOC05_12520], nitrilotriacetate monooxygenase [AOC05_10370] and aliphatic amidase amiE [AOC05_16895].

Furthermore, two genes involved in the production of urease were also identified in the genome of 10.1601/nm.20084 strain R3.8, urease alpha subunit [AOC05_06080] and urease gamma subunit [AOC05_18490]). Urease is important in catalyzing one of the metabolic pathways involved in microbial-induced calcite precipitation. MICP is a promising approach in the containment of heavy metals such as lead and cadmium in contaminated soils [23, 24].

Various other xenobiotic biodegradation genes and pathways of 10.1601/nm.20084 strain R3.8 are available from the PATRIC server.

## Genes with chitinolytic and *N*-acetylglucosamine utilization properties

Chitinase is a biotechnologically-important enzyme widely used in waste management industries for the degradation of chitinous waste into simpler depolymerized substances [25], in agricultural industries for engineering of transgenic crops with resistance to fungal infection [26] and in healthcare industries for the therapeutic treatment of fungal infections [25, 27]. A range of recent tudies have identified and characterized novel cold-active chitinase enzymes with higher catalytic efficiency at low temperatures [28–31].

The full chitinolytic potential of 10.1601/nm.20084 strain R3.8 was also identified here, with various genes involved in chitin and *N*-acetylglucosamine utilization being identified, including beta-hexosaminidase (EC 3.2.1.52) [AOC05_10575, AOC05_02140], eukaryotic type *N*-acetylglucosamine kinase (EC 2.7.1.59) [AOC05_12050], PTS system, *N*-acetylglucosamine (EC 2.7.1.69) [AOC05_12345], *N*-acetylglucosamine-6-phosphate deacetylase (EC 3.5.1.25) [AOC05_15120], eukaryotic type *N*-acetylglucosamine kinase (EC 2.7.1.59) [AOC05_15120], chitinase (EC 3.2.1.14) [AOC05_02965], *N*-acetyl-glucosamine kinase 2, ROK family (EC 2.7.1.59) [AOC05_03295], transcriptional regulator of *N*-acetylglucosamine utilization, GntR family [AOC05_07215] and glucosamine-6-phosphate deaminase (EC 3.5.99.6) [AOC05_10045].

## Potential plant growth promoting properties

Application of psychrotrophic PGP bacteria to vegetation can promote growth and improve cold tolerance of crops [32]. From the RAST analysis, a total of 22 PGP genes were identified in the genome of 10.1601/nm.20084 strain R3.8. The genes that are involved in the glutamine synthetase (GS)-glutamate synthase (GOGAT) pathway of bacterial ammonia assimilation are glutamine synthetase (GS) [AOC05_04790] and glutamate synthase (GOGAT) [AOC05_05585]. Full details of these genes, including nitrogen regulatory protein [AOC05_18400] and ammonium transporter [AOC05_09335] are available in Additional file 1: Table S1. Furthermore, five genes involved in plant hormone biosynthesis, indole acetic acid biosynthesis, including monoamine oxidase [AOC05_17530], tryptophan synthase [AOC05_05570, AOC05_05575], anthranilate phosphoribosyltransferase [AOC05_07190] and *N*-(5′-phosphoribosyl) anthranilate isomerase [AOC05_12350] were identified. Production of bacterial IAA is important to assist plants to overcome abiotic stresses and inhibitory compounds, and thus contributes to plant growth stimulation [33]. Several other PGP-relevant genes involved in trehalose synthesis [AOC05_15010, AOC05_00140, AOC05_00145, AOC05_00495, and AOC05_00500] and involved in spermidine synthesis [AOC05_16565] were also identified in the genome.

## Conclusions

We report the complete genome sequence of 10.1601/nm.20084 strain R3.8 that was originally isolated from the soil collected from Rothera Point, Adelaide Island, maritime Antarctica. The strain was sequenced to explore its biotechnological potential. By analyzing the complete genome of 10.1601/nm.20084 strain R3, we identified genes involved in xenobiotic biodegradation and metabolism, and chitin utilization, as well as genes that potentially promote plant growth. Further comparative genomic studies with related isolates together with functional studies will provide better understanding of the potential biotechnological value of this strain.

## Additional file

Additional file 1:
**Table S1.** Genes involved in chitin degradation identified from RAST analysis. (DOCX 18 kb)

## Abbreviations

HGAP: Hierarchical Genome Assembly Process
IAA: Indole acetic acid
MICP: Microbial-induced calcite precipitation
MIGS: Minimum Information about the Genome Sequences
PATRIC: Pathosystem Resource Integration Center
PGP: Plant Growth Promoting
RAST: Rapid Prokaryotic Genome Annotation
SMRT: Single Molecule Real Time
