# Supplementary material for: Complete genome of Arthrobacter alpinus strain R3.8, bioremediation potential unraveled with genomic analysis
Source: Stand Genomic Sci. 2017 Sep 6;12:52. doi: 10.1186/s40793-017-0264-0 (PMC5586057; doi:10.1186/s40793-017-0264-0)
Supplement: Additional file 1: — Table S1. Genes involved in chitin degradation identified from RAST analysis. (DOCX 18 kb) [file 40793_2017_264_MOESM1_ESM.docx]

**Additional file 1: Table S1.** Genes involved in chitin degradation identified from RAST analysis

| **Feature ID** | **Start** | **Stop** | **Length (bp)** | | **Function** | **Subsystem** |
| --- | --- | --- | --- | --- | --- | --- |
| **Chitin Degradation** | | | | | | |
| fig\|6666666.133317.peg.98 | 99163 | 97880 | 1284 | Beta-hexosaminidase (EC 3.2.1.52) | | Chitin and N-acetylglucosamine utilization |
| fig\|6666666.133317.peg.393 | 430502 | 429570 | 933 | N-acetylglucosamine kinase of eukaryotic type (EC 2.7.1.59) | | Chitin and N-acetylglucosamine utilization |
| fig\|6666666.133317.peg.461 | 496195 | 494645 | 1551 | PTS system, N-acetylglucosamine-specific IIA component (EC 2.7.1.69) / PTS system, N-acetylglucosamine-specific IIB component (EC 2.7.1.69) / PTS system, N-acetylglucosamine-specific IIC component (EC 2.7.1.69) | | Chitin and N-acetylglucosamine utilization, Chitin and N-acetylglucosamine utilization, Chitin and N-acetylglucosamine utilization, Sialic Acid Metabolism, Sialic Acid Metabolism, Sialic Acid Metabolism |
| fig\|6666666.133317.peg.627 | 664590 | 665975 | 1386 | N-acetylglucosamine-6-phosphate deacetylase (EC 3.5.1.25) | | Chitin and N-acetylglucosamine utilization, Sialic Acid Metabolism |
| fig\|6666666.133317.peg.1026 | 1087624 | 1088634 | 1011 | N-acetylglucosamine kinase of eukaryotic type (EC 2.7.1.59) | | Chitin and N-acetylglucosamine utilization |
| fig\|6666666.133317.peg.2160 | 2291942 | 2290413 | 1530 | Beta-hexosaminidase (EC 3.2.1.52) | | Chitin and N-acetylglucosamine utilization |
| fig\|6666666.133317.peg.2299 | 2450202 | 2445454 | 4749 | Chitinase (EC 3.2.1.14) | | Chitin and N-acetylglucosamine utilization |
| fig\|6666666.133317.peg.2325 | 2477856 | 2479265 | 1410 | Chitinase (EC 3.2.1.14) | | Chitin and N-acetylglucosamine utilization |
| fig\|6666666.133317.peg.2328 | 2488027 | 2483738 | 4290 | Chitinase (EC 3.2.1.14) | | Chitin and N-acetylglucosamine utilization |
| fig\|6666666.133317.peg.2394 | 2579259 | 2580227 | 969 | Predicted N-acetyl-glucosamine kinase 2, ROK family (EC 2.7.1.59) | | Chitin and N-acetylglucosamine utilization |
| fig\|6666666.133317.peg.3189 | 3422969 | 3422190 | 780 | Predicted transcriptional regulator of N-Acetylglucosamine utilization, GntR family | | Chitin and N-acetylglucosamine utilization |
| fig\|6666666.133317.peg.3770 | 4027266 | 4026484 | 783 | Glucosamine-6-phosphate deaminase (EC 3.5.99.6) | | Chitin and N-acetylglucosamine utilization, Sialic Acid Metabolism |
| fig\|6666666.133317.peg.3779 | 4036711 | 4037961 | 1251 | N-acetylglucosamine-6-phosphate deacetylase (EC 3.5.1.25) | | Chitin and N-acetylglucosamine utilization, Sialic Acid Metabolism |
